# The progress of medication-related osteonecrosis of the jaw with conservative initial treatment: A 12-year retrospective study of 129 patients

**DOI:** 10.1016/j.bonr.2021.101072

**Published:** 2021-04-21

**Authors:** Nobuyuki Kaibuchi, Keika Hoshi, Ayame Yamazaki, Noriko Miyamoto-Sangu, Yuichi Akagi, Toshihiro Okamoto

**Affiliations:** a Department of Oral and Maxillofacial Surgery, Tokyo Women's Medical University School of Medicine, 8-1 Kawada-cho, Shinjuku-ku, Tokyo 162-8666, Japan; b Institute of Advanced Biomedical Engineering and Science, Tokyo Women's Medical University (TWIns), 8-1 Kawada-cho, Shinjuku-ku, Tokyo 162-8666, Japan; c National Institute of Public Health, Center for Public Health Informatics, 2-3-6 Minami, Wako, Saitama 351-0197, Japan; d Department of Hygiene, Kitasato University School of Medicine, 1-15-1 Kitasato, Minami-ku, Sagamihara, Kanagawa 252-0373, Japan

**Keywords:** MRONJ, medication-related osteonecrosis of the jaw, OR, odds ratio, CI, confidence interval, BMA, bone modifying agents, BP, bisphosphonate, Dmab, denosumab, Osteonecrosis of the jaw, Bisphosphonate, Denosumab, Conservative treatment, Sequestrum, Retrospective study

## Abstract

This retrospective study aimed to examine the course and prognosis of medication-related osteonecrosis of the jaw (MRONJ) initially treated conservatively and the effects of various factors affecting treatment outcomes. We evaluated 129 patients with MRONJ between January 2008 and December 2018 at a university hospital. The factors examined included sex, age, stage of MRONJ (1–3), type of bone modifying agents (bisphosphonate or denosumab), primary disease (osteoporosis or malignant tumor), medical history (diabetes and rheumatoid arthritis), use of corticosteroids, the trigger of MRONJ (teeth extraction or others), and separation of sequestrum, using logistic regression analysis. Patients with MRONJ were treated conservatively as the initial treatment in accordance with the position paper of the American Association of Oral and Maxillofacial Surgeons. Of the 129 patients, 59 (45.7%) were cured, and the condition of 70 (54.3%) remained unchanged or worsened. The overall cure rates at 12, 36, and 60 months were 25.8%, 50.8%, and 72.4% respectively. The cure rate of stage 1 was lower than that of stages 2 and 3 at 80 months. In multivariate analysis, it was found that 37 (64.9%) of 57 patients with osteoporosis as a primary disease were cured (odds ratio [OR], 7.7; 95% confidence interval [CI], 2.4–24.4). In addition, 40 (69.0%) of 58 patients with separation of sequestrum were cured (OR, 8.9; 95% CI, 3.4–23.5). The cure rate was significantly higher in patients with osteoporosis than in those with cancer when the treatment outcomes of primary disease were compared using the Kaplan-Meier method (*p* < 0.01). It was also significantly higher in patients who had separation of sequestrum than in those who did not (*p* < 0.05). Our results suggest that primary disease and separation of sequestrum were associated with favorable outcomes in patients with MRONJ initially treated conservatively. MRONJ had a poor prognosis with conventional treatment carried according to the stage of the disease. This was especially prominent when conservative treatment was employed for mild cases.

## Highlights

• Of the 129 patients with MRONJ, 59 (45.7%) were cured, and the condition of 70 (54.3%) remained unchanged or worsened.
• The cure rate of stage 1 was lower than that of stages 2 and 3 at 80 months.
• Primary disease and separation of sequestrum were associated with outcomes in MRONJ initially treated conservatively.

## 1 Introduction

Medication-related osteonecrosis of the jaw (MRONJ) is a common adverse effect of anti-resorptive agents and angiogenesis inhibitors. There is still no definitive treatment for MRONJ, and determining a treatment strategy remains a challenge (Ruggiero et al., 2014). Whether MRONJ should be treated surgically or conservatively remains controversial. Although surgical procedures have been reported to have a better prognosis than conservative treatment, many patients do not opt for surgery because of the complexity of their medical problem or socio-economic background (Hayashida et al., 2020). Furthermore, definite criteria for the timing of surgery have not been established. Surgery as an initial treatment may be unnecessary in some patients who may be cured with conservative treatment. It is important to study the course of patients treated conservatively to determine the timing of surgery. In this study, we examined the course and prognosis of patients treated conservatively as the initial treatment and the effects of various factors affecting treatment outcomes.

## 2 Methods

### 2.1 Study design

**Fig. 1:**
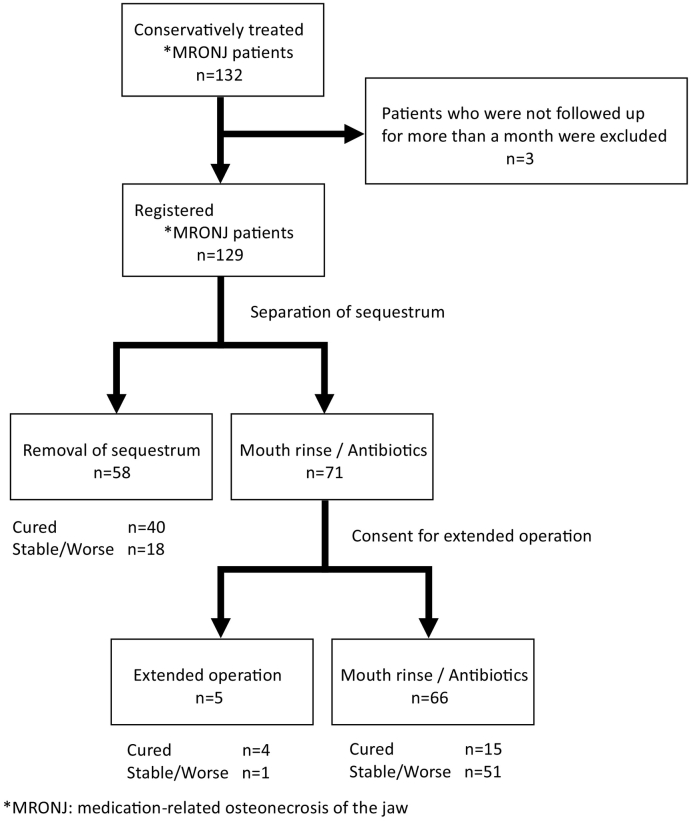
Treatment strategy and outcomes.

This was a single-center retrospective cohort study to evaluate the course, prognosis, and various prognostic factors in MRONJ patients who were treated conservatively as an initial treatment. A total of 132 patients with MRONJ were treated at the Tokyo Women's Medical University Hospital between January 2008 and December 2018. Three patients who were not followed up for more than a month after diagnosis and initial treatment were excluded from this study (Fig. 1).

### 2.2 Characteristics of MRONJ patients

A summary of 129 patients with MRONJ is presented in Table 1. There were 27 men and 102 women, with 79% of the total number being females. The median age was 76 years and the mean age was 74.3 ± 11.5 years. BP was administered to 89 patients and Dmab to 40 patients. The primary disease was malignant tumors in 72 patients and osteoporosis in 57 patients. Stage 2 MRONJ was exhibited in 76 patients and was the most common stage (Table 1).

**Table 1:** Characteristics of 129 MRONJ patients.

	Number (%)
Number of patients	129
Gender	
Male	27 (21)
Female	102 (79)
Age (years)	
Range(min-max) (years)	42–99
Mean ± SD	74.3 ± 11.5
Stage of MRONJ	
1	26 (20)
2	76 (59)
3	27 (21)
Type of BMA	
Bisphosphonate	89 (69)
Denosumab	40 (31)
Primary disease	
Malignant tumor	72 (56)
Osteoporosis	57 (44)
Diabetes	
Yes	25 (19)
No	104 (81)
Rheumatoid arthritis	
Yes	32 (25)
No	97 (75)
Use of corticosteroid	
Yes	48 (37)
No	81 (63)
Trigger	
Tooth extraction	58 (45)
Others	71 (55)
Separation of sequestrum	
Yes	58 (45)
No	71 (55)
Surgical treatment	
Yes	63 (49)
No	66 (51)

MRONJ: medication-related osteonecrosis of the jaw; BMA: bone modifying agents.

### 2.3 Variables

The factors that were examined based on the patient's medical records included: sex, age, stage of MRONJ (1–3), type of bone modifying agent (BMA) [bisphosphonate (BP) or denosumab (Dmab)] administered, primary disease (osteoporosis or malignant tumor), medical history (diabetes and rheumatoid arthritis), use of corticosteroids, the trigger of MRONJ (teeth extraction or others), separation of sequestrum, and surgical treatment (extended operation and sequestrectomy).

### 2.4 Treatment strategy

**Fig. 2:**
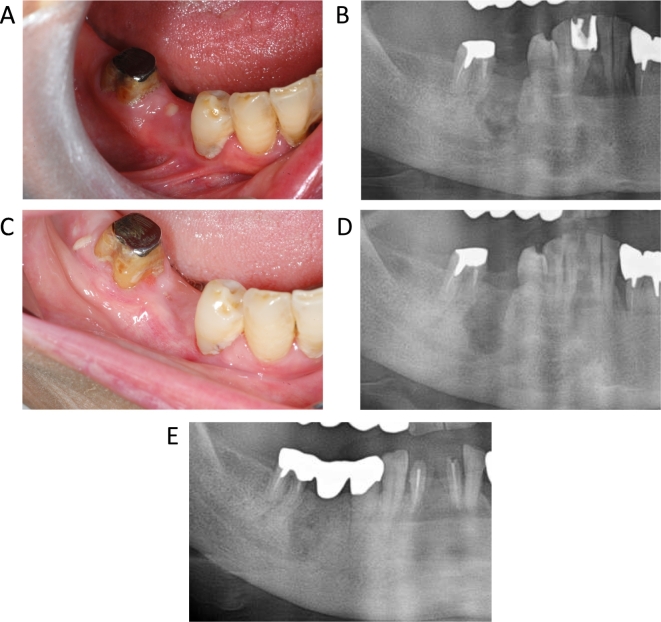
A representative case of a 79-year-old woman diagnosed with stage 2 medication-related osteonecrosis of the jaw (MRONJ) with separation of sequestrum. A: Pus and fistula in gingiva after second premolar extraction. Alveolar bone can be probed through the fistula. B: Separation of sequestrum was observed on panoramic X-ray. C: Complete mucosal wound healing was observed after sequestrectomy. D: Panoramic X-ray after sequestrectomy. E: Two years after surgery, bone regeneration and no recurrence were observed.

The position paper of the American Association of Oral and Maxillofacial Surgeons (Ruggiero et al., 2014) and Japanese position paper (Yoneda et al., 2017) recommended conservative treatment for stage 1–2 of MRONJ. Thus, stages 1–2 were treated with mouth rinse (saline or povidone‑iodine) and antibiotics (penicillin compounds, cephem compounds, or macrolide compounds) as a part of the conservative treatment in our department. In addition, stage 3 patients who could not be operated on for reasons such as their general condition or refusal to surgery were conservatively treated. In all patients, sequestrectomy was performed immediately after separation of the sequestrum. Separation of the sequestrum was defined as the presence of clinically necrotic bone wobble or the presence of bone-free from the jawbone on X-ray or Computed Tomography (Fig. 2). In patients where necrosis was unchanged or aggravated, an extended operation was considered only after a month of conservative treatment (Fig. 1).

### 2.5 Outcome

The primary outcome of the study was the cure rate of MRONJ. Cure was defined as the complete closure of the mucosal wound. The outcome was assessed by visual inspection and palpation using dental instruments.

### 2.6 Statistical analysis

All statistical analyses were performed using JMP Pro 14.0.0 (SAS, Cary, NC, USA). The factors and treatment outcomes were analyzed in all 129 patients using the Kaplan-Meier method and log-rank test. Multivariate analyses of the factors for treatment outcomes were performed using logistic regression analysis with sex, age, stage of MRONJ, type of BMA, primary disease, medical history (diabetes and rheumatoid arthritis), use of corticosteroids, the trigger of MRONJ, and separation of sequestrum as adjusted variables. ORs and 95% CI for each factor were calculated. The association of each factor with the separation of sequestrum was analyzed using Student's *t*-test or Pearson's chi-square test. Statistical significance was set at *p* < 0.05.

### 2.7 Ethics

This study was approved by the Institutional Review Board (Ethics Committee of Tokyo Women's Medical University Hospital, No. 5047).

## 3 Results

### 3.1 Overall treatment outcomes

**Fig. 3:**
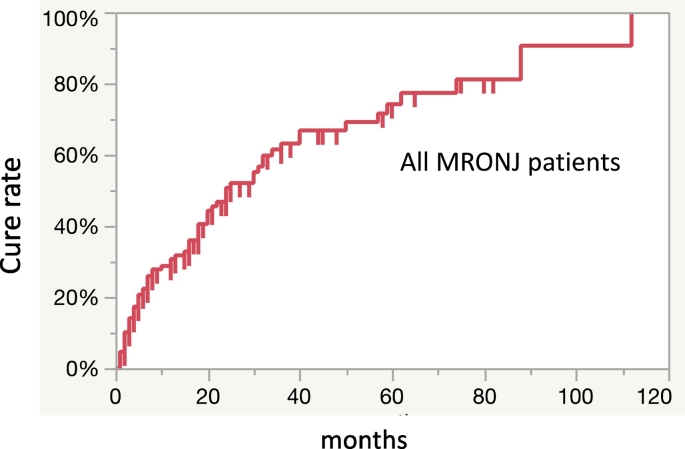
The overall cure rate of 129 MRONJ patients at 12, 36, 60 months using the Kaplan-Meier method was 25.8%, 50.8%, 72.4%.

**Fig. 4:**
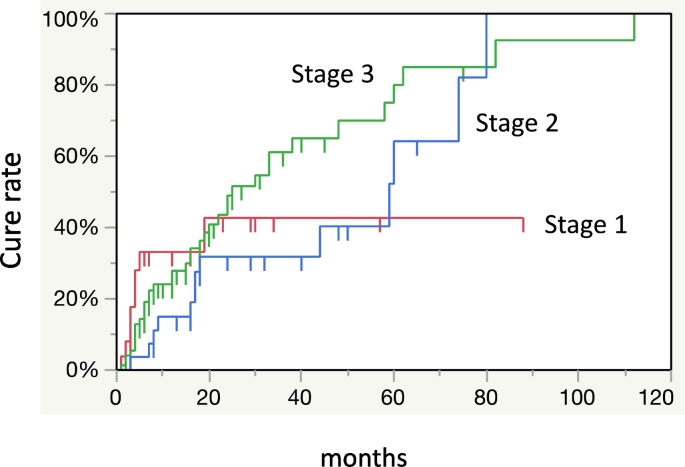
The cure rate by stage as defined in the position paper was calculated. The cure rate of stage 1 was lower than that of stage 2 and 3 at 80 months.

Separation of sequestrum was observed in 58 (45.0%) of 129 patients with MRONJ treated conservatively as an initial treatment (Fig. 1). Forty (70.0%) of 58 patients were cured after sequestrectomy. Extended operations were performed in five patients who did not have separation of the sequestrum after informed consent. Overall, 59 (45.7%) patients were deemed cured and for 70 (54.3%), the condition remained unchanged or worsened. The overall cure rates at 12, 36, and 60 months were 25.8%, 50.8%, and 72.4% respectively (Fig. 3). The cure rate of stage 1 at 80 months was lower than that of stages 2 and 3 (Fig. 4).

### 3.2 Factors affecting treatment outcome

**Fig. 5:**
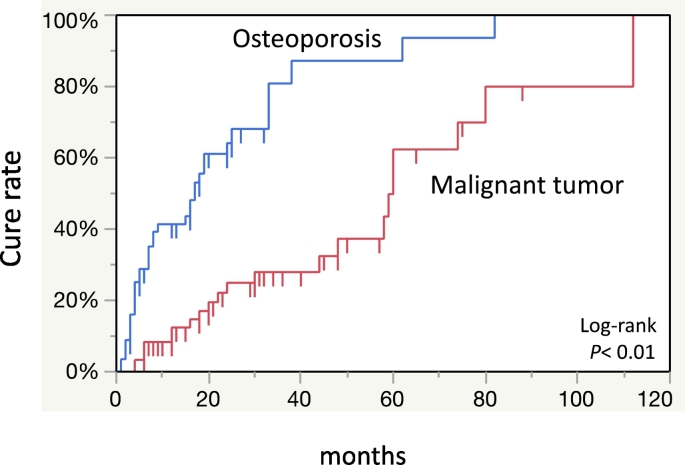
The cure rate was significantly lower in patients with cancer than those with osteoporosis.

**Fig. 6:**
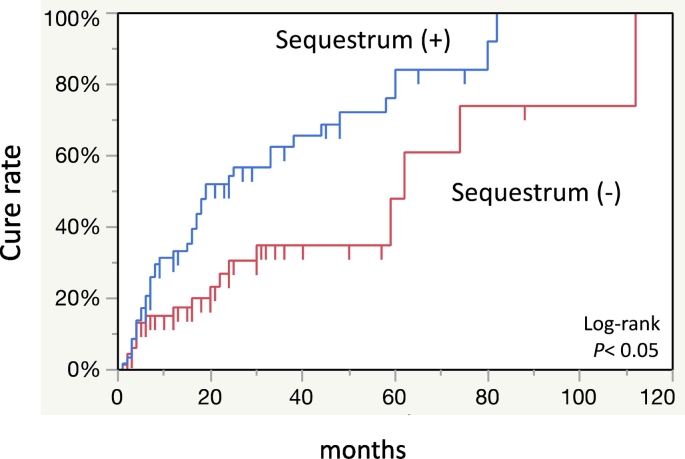
The cure rate was significantly higher in patients who had separation of sequestrum compared to those who did not.

Multivariate analysis was performed to examine the effects of various factors on the treatment outcomes. This showed that primary disease and separation of sequestrum were significantly associated with treatment outcomes (Table 2). Among patients with osteoporosis as the primary disease, 37 (64.9%) out of 57 patients were cured (OR: 7.7; 95% CI: 2.4–24.4). In addition, 40 (69.0%) out of 58 patients with separation of sequestrum were cured (OR, 8.9; 95% CI, 3.4–23.5). Univariate analysis of the presence or absence of sequestrum separation revealed no significant difference between the two groups (Table 3). The cure rate was significantly higher in patients with osteoporosis than in those with cancers when the treatment outcomes due to primary disease were compared using the Kaplan-Meier method (*p* < 0.01) (Fig. 5). The cure rate was significantly higher in patients who had separation of sequestrum than in those who did not (*p* < 0.05) (Fig. 6).

**Table 2:** Multivariate analysis of various factors affecting treatment outcome.

Factors		OR	95% CI	*p* value
Age (years)		0.98	0.9–1.0	0.366
Sex	Male/female	2	0.6–6.6	0.242
Stage	2/1	3.3	0.9–11.5	0.066
	3/1	2.2	0.5–9.6	0.298
Type of BMA	Dmab/BP	2.9	0.9–8.8	0.056
Primary disease	Osteoporosis/malignant tumor	7.7	2.4–24.4	<0.001
Diabetes	(+)/(−)	1.2	0.4–3.6	0.697
Rheumatoid arthritis	(+)/(−)	1.9	0.5–7.2	0.355
Use of corticosteroid	(+)/(−)	0.6	0.2–1.9	0.381
Trigger	Other/tooth extraction	1.4	0.6–3.5	0.451
Separation of sequestrum	(+)/(−)	8.9	3.4–23.5	<0.001

OR: odds ratio, CI: confidence interval.

**Table 3:** Comparison of various factors in presence or absence of separation of sequestrum.

Separation of sequestrum	Yes	No	*p* value
Gender			
Male	8	19	
Female	50	52	0.084
Age (years)			
Range(min-max) (years)	55–99	42–99	0.059
Mean ± SD	76.5 ± 10.3	72.6 ± 12.3	
Stage of MRONJ			
1	10	16	0.232
2	32	44	
3	16	11	
Type of BMA			
Bisphosphonate	43	46	0.253
Denosumab	15	25	
Primary disease			
Malignant tumor	27	45	0.055
Osteoporosis	31	26	
Diabetes			
Yes	11	14	0.914
No	47	57	
Rheumatoid arthritis			
Yes	16	16	0.509
No	42	55	
Use of corticosteroid			
Yes	23	25	0.604
No	35	46	
Trigger			
Tooth extraction	31	27	0.080
Others	27	44	

MRONJ: medication-related osteonecrosis of the jaw; BMA: bone modifying agents.

## 4 Discussion

Various departments involved in cancer or bone diseases such as oral and maxillofacial surgery and dentistry are unclear on how to determine a treatment strategy for MRONJ. In a position paper, a treatment strategy is recommended for each stage of MRONJ (Ruggiero et al., 2014). We treated MRONJ according to the position paper. However, only 59 (45.7%) out of the 129 patients were eventually cured (Fig. 1). First, the stage classification in the position paper may not correctly represent the clinical symptoms and pathophysiology of MRONJ. In our retrospective study, our results showed that the cure rate did not decrease as the stage progressed (Fig. 4). Recently, several reports have recommended surgical treatment as opposed to conservative treatment in mild cases (stages 1 and 2) as the initial treatment (Hayashida et al., 2020; Rupel et al., 2014; Fliefel et al., 2015; Khan et al., 2015). However, there is a lack of clarity regarding detailed surgical procedures and the timing of operations. The cure rate of surgical treatment of MRONJ ranges from 60% to 80% (Hayashida et al., 2020; Rupel et al., 2014; Fliefel et al., 2015; Hayashida et al., 2017). A multicenter retrospective study concluded that half of the surgical treatment cases of MRONJ due to cancer and treated with BMA were not completely cured (Hayashida et al., 2017). Since patients in stages 1 and 2 of MRONJ are often unaware of the symptoms, it may be difficult to obtain their consent for surgery that may have uncertain outcomes. However, some cases require surgical treatment to achieve a radical cure. Therefore, we examined the prognosis of cases treated conservatively as the initial treatment to establish the timing of operation for MRONJ. Our results showed that there was no tendency to peak at any point and the duration of conservative treatment was not clear (Fig. 3).

Multivariate analysis revealed that separation of sequestrum and primary disease were factors affecting treatment outcome (Table 2). In addition, there were significant differences in these two factors in the Kaplan-Meier study of cure rates (Fig. 5, Fig. 6). We also noted a significant difference in cure rates between primary diseases of cancer and osteoporosis (Hayashida et al., 2020). The key point of surgery for MRONJ may be the decision on resection margins and how to handle the wound of the bone after resection. In light of the above, MRONJ patients with separation of sequestrum have clear resection margins, and early wound closure can be expected due to mucosal penetration. Aghaloo et al. reported that epithelial rimming occurs around the separated sequetrum which subsequently exfoliates through the soft tissue over time (Aghaloo et al., 2011). Moreover, Soundia et al. reported that sequestration on radiographs is a predictor of future bone exposure in patients with stage 0 MRONJ (Soundia et al., 2018). Also, Hadaya et al. reported that sequestration is a key determinant in healing of MRONJ and only 5% of the patients with separation of sequestrum had recurrence (Hadaya et al., 2018). However, the background of separation of sequestrum in patients is not clear. Although not significant, there was more separation of sequestrum in patients with osteoporosis than in patients with malignancy (Table 3). There are some case reports in which separation of sequestrum was observed after administration of teriparatide (Cheung and SeemanCase, 2010; Zushi et al., 2017; Doh et al., 2015; Lee et al., 2011). Moreover, a randomized control trial showed that the administration of teriparatide significantly promoted bone healing in MRONJ (Sim et al., 2020). However, the underlying mechanism remains unclear.

This study was a retrospective cohort study and had several limitations, such as the surgical methods were not always consistent and several pieces of information were not available. Therefore, it is not possible to prove causality and generalization of the results may be difficult. We believe that prospective studies are needed to draw more definite conclusions.

## 5 Conclusions

MRONJ had a poor prognosis with conventional treatments by stage. Although many studies have investigated novel treatments for MRONJ (Freiberger et al., 2012; Curi et al., 2011; Favia et al., 2018; Park et al., 2017; Heifetz-Li et al., 2019; Kaibuchi et al., 2019; Kaibuchi et al., 2016), they are not widely accepted. Further research is needed to establish treatment strategies for MRONJ.

## Funding

This work was partially supported by the 10.13039/501100001691Japan Society for the Promotion of Science KAKENHI, Grant numbers 18K17181 and 15K11224.

## CRediT authorship contribution statement

**Nobuyuki Kaibuchi:** Conceptualization, Methodology, Software, Formal analysis, Writing – original draft. **Keika Hoshi:** Software, Formal analysis, Supervision. **Ayame Yamazaki:** Data curation. **Noriko Miyamoto-Sangu:** Data curation. **Yuichi Akagi:** Data curation. **Toshihiro Okamoto:** Supervision, Writing – review & editing.

## Declaration of competing interest

The authors declare that they have no known competing financial interests or personal relationships that could have appeared to influence the work reported in this paper.
